# [BMP]^+^[BF_4_]^−^-Modified CsPbI_1.2_Br_1.8_ Solar Cells with Improved Efficiency and Suppressed Photoinduced Phase Segregation

**DOI:** 10.3390/molecules29071476

**Published:** 2024-03-26

**Authors:** Haixia Xie, Lei Li, Jiawei Zhang, Yihao Zhang, Yong Pan, Jie Xu, Xingtian Yin, Wenxiu Que

**Affiliations:** 1 School of Science, Xi’an University of Architecture and Technology, Xi’an 710055, China; hao@xauat.edu.cn (Y.Z.); panyong@xauat.edu.cn (Y.P.); jiexu@xauat.edu.cn (J.X.); 2 State Key Laboratory for Strength and Vibration of Mechanical Structures, School of Aerospace Engineering, Xi’an Jiaotong University, Xi’an 710049, China; 3 Electronic Materials Research Laboratory, Key Laboratory of the Ministry of Education, International Center for Dielectric Research, Shaanxi Engineering Research Center of Advanced Energy Materials and Devices, School of Electronic Science and Engineering, Xi’an Jiaotong University, Xi’an 710049, China; xjtu_li@stu.xjtu.edu.cn (L.L.); zjw3026@stu.xjtu.edu.cn (J.Z.); wxque@xjtu.edu.cn (W.Q.)

**Keywords:** CsPbI_1.2_Br_1.8_, phase segregation, Br-mixed halogen perovskites, perovskite solar cell, stability

## Abstract

With the rapid progress in a power conversion efficiency reaching up to 26.1%, which is among the highest efficiency for single-junction solar cells, organic–inorganic hybrid perovskite solar cells have become a research focus in photovoltaic technology all over the world, while the instability of these perovskite solar cells, due to the decomposition of its unstable organic components, has restricted the development of all-inorganic perovskite solar cells. In recent years, Br-mixed halogen all-inorganic perovskites (CsPbI3−xBrx) have aroused great interests due to their ability to balance the band gap and phase stability of pure CsPbX3. However, the photoinduced phase segregation in lead mixed halide perovskites is still a big burden on their practical industrial production and commercialization. Here, we demonstrate inhibited photoinduced phase segregation all-inorganic CsPbI1.2Br1.8 films and their corresponding perovskite solar cells by incorporating a 1-butyl-1-methylpiperidinium tetrafluoroborate ([BMP]+[BF4]−) compound into the CsPbI1.2Br1.8 films. Then, its effect on the perovskite films and the corresponding hole transport layer-free CsPbI1.2Br1.8 solar cells with carbon electrodes under light is investigated. With a prolonged time added to the reduced phase segregation terminal, this additive shows an inhibitory effect on the photoinduced phase segregation phenomenon for perovskite films and devices with enhanced cell efficiency. Our study reveals an efficient and simple route that suppresses photoinduced phase segregation in cesium lead mixed halide perovskite solar cells with enhanced efficiency.

## 1. Introduction

Organic–inorganic hybrid perovskite solar cells have gained significant attention due to their exceptional performance, tunable bandgap, high light-absorption coefficient, low cost and versatile applications, making them highly valuable for commercial use. The certified power conversion efficiency (PCE) of perovskite solar cells has rocketed up to 26.1% at a lab-scale at present, from 3.8% in 2009, which is comparable to that of well-developed single-junction silicon solar cells [1,2,3,4,5]. Currently, most of the perovskite solar cells with high PCEs over 20% are constructed from organic–inorganic hybrid perovskite materials listed as ABX_3_, where usually A is one or more monovalent methylamine ions such as methylamine ions (CH_3_NH_3_^+^, referred to as MA^+^), ethylamine ions (CH_3_CH_2_NH_3_^+^, referred to as EA^+^), formamidine cations (NH = CHNH_3_^+^, referred to as FA^+^), etc. B is one or more of a divalent metal cation of a carbon family such as lead (Pb^2+^), tin (Sn^2+^), germanium (Ge^2+^) cations), etc. And X is one or more of the halogen ions such as Cl^−^, Br^−^, and I^−^ [3,6,7,8]. However, the long-term stability issues of organic–inorganic hybrid perovskite materials under moisture, oxygen, thermal external force and even persistent light are still big burdens for their future application. These issues are caused by the inevitable phase segregation, ion migration, and crystal decomposition in the volatile nature of the organic components [9,10,11,12,13].

To address the issue of instability, it is suggested that the organic cations be replaced with all inorganic Cs^+^ to create CsPbX_3_ (X = I^−^, Br^−^, Cl^−^ or their mixtures) [14,15,16,17,18]. CsPbX_3_ typically has four types of crystal structures, including the cubic structure (α-, Pm3m), the tetragonal structure (β-, P4/mbm), the orthorhombic structure (γ-, Pbnm), and a non-perovskite structure (δ-, Pnma) [19]. Research into CsPbX_3_ perovskite solar cells started in 2014, when Choi et al. attempted to improve the performance of perovskite solar cells by removing MA^+^ from Cs_x_MA_1−x_PbI_3_ solar cells. All inorganic δ-CsPbI_3_ (*E*_g_ = 2.82 eV) perovskite solar cells showed a PCE of only 0.09% [20]. In 2015, Gary Hodes et al. first fully investigated whether the organic cation was necessary to obtain devices with high photovoltaic performance. They found that the fabricated inorganic CsPbBr_3_ perovskite solar cells, which performed equally as well as the organic ones, were much more temperature stable than the hybrid analogues. This study conducted useful comparative studies between hybrid organic–inorganic and all-inorganic perovskite materials [21]. Shortly thereafter, Snaith et al. reported on inorganic CsPbI_3_ (*E*_g_ = 1.73 eV) perovskite solar cells. The current density–voltage (*J*-*V*) curve showed an efficiency of up to 2.9%, which was more stable than that of the hybrid organic–inorganic perovskite solar cells [22]. In 2017, Snaith’s group used vacuum-based vapor deposition for alternating very thin layers of CsI and PbI_2_ and obtained a stabilized PCE of 7.8 %, compared to the 4.3 % for spin-coated CsPbI_3_ solar cells. The main improvement in vapor-deposited perovskite films was attributed to the much longer carrier lifetimes (>10 μs) compared to the spin-coated ones and their presumed lower trap densities [23]. CsPbX_3_ perovskite solar cells then entered a period of rapid development through various optimization strategies such as additive engineering, interfacial engineering and precursor solution engineering. Cs-based perovskite solar cells have achieved a high PCE of approximately 21% with a high stability against humidity and heat [24]. From this progress, CsPbX_3_ materials are emerging as a new research area and will play a significant role in the photovoltaic field in the near future.

Within the CsPbX_3_ family, CsPbBr_3_ exhibits exceptional moisture and thermal stability. However, due to its large band gap of approximately 2.3 eV, it can only absorb light from the UV region [25]. Compared to other CsPbX_3_ compounds, cubic CsPbI_3_ has the narrowest band gap of 1.73 eV, which allows for a wider range of light absorption. However, its application is limited due to the phase instability caused by transitions between the desirable photoactive perovskite black phase (*α*-CsPbI_3_, cubic Pm3m) and the undesirable non-perovskite yellow phase (*δ*-CsPbI_3_, orthorhombic Pnma), although the transition to the yellow phase is reversible [26,27]. Br-mixed halogen perovskites (CsPbI_3−x_Br_x_) are of great interest due to their ability to balance the band gap and phase stability of CsPbX_3_. Additionally, the band gap of CsPbI_3−x_Br_x_ can be precisely tuned from ~1.2 to 2.4 eV by adjusting the value of x. This is because the changes in the Br^−^ and Cl^−^ ratio can monotonically shift the absorption onset and lead to lattice transitions for perovskite, resulting an alteration in the photonic bandgaps and, thus, making them promising candidates for the sub-cells of tandem solar cells [17,28,29,30].

Unexpectedly, some researchers have found that mixed halide perovskites suffer from intrinsic photoinduced halide segregation, with more bromide replacing iodide ions [31,32,33,34]. And, numerous studies have demonstrated that the phenomenon of photoinduced phase segregation decreases the *V*_oc_ of the device and significantly impacts its photoelectric performance [35,36]. Eric T. Hoke et al. are the pioneers who discovered the photoinduced phase segregation in mixed halide perovskite films in 2015. They found that when 0.2 < x < 1, the photoluminescence (PL) spectra of the MAPb(Br_x_I_1−x_)_3_ films formed a new emission peak at 1.68 eV and a redshift phenomenon occurred, indicating photoinduced phase segregation. Surprisingly, these changes in photoinduced phase segregation were completely reversible by switching between light and dark [31]. In 2017, Yi-Bing Cheng et al. observed iodide-rich phase segregation near grain boundaries in nanoscale all-inorganic CsPbIBr_2_ films. The authors found that iodide segregation can occur due to the immiscibility between iodine and bromine, which exacerbates ion migration and hysteresis, thereby affecting the photoelectric performance of the corresponding devices [32]. However, the exact mechanism by which the photoinduced phase segregation phenomenon affects the *V*_oc_ of the devices is not clear. In addition, since the photoinduced phase segregation phenomenon destabilizes the perovskite solar cells, leading to a decrease in the PCE and *V*_oc_ of the devices, researchers have done a great deal of research work to inhibit and mitigate the occurrence of the photoinduced phase segregation phenomenon in perovskite solar cell devices. For example, Yue Hao et al. proposed a simple and effective strategy to inhibit the photoinduced phase segregation in CsPbIBr_2_ films by modifying their crystalline grains with poly(methyl methacrylate) (PMMA). As a result, the carbon electrode-based CsPbIBr_2_ PSC exhibited a more suppressed photocurrent hysteresis, coupled with an excellent PCE of 9.21% and a high *V*_oc_ of 1.307 V. Their work not only provided a new avenue to address the general halide phase segregation issue of CsPbIBr_2_ materials, but also provided guidance to achieve the superior performance of opto-electronic devices [37]. Very recently, Wei Li et al. unveiled the impact of phase segregation in Cs_0.17_FA_0.83_Pb(I_0.80_Br_0.20_)_3_ films with a bandgap of 1.67 eV through a photoconductive atomic force microscopy. By testing the *I*-*V* curves at both grain boundaries and grain interiors with nanoscopic resolution, they identified that iodide-rich phases primarily segregated at defect-enriched grain boundaries under continuous illumination, causing a more significant local open-circuit voltage (*V*_OC_) decrease than that occurring at grain interiors [38].

In this work, all inorganic CsPbI_1.2_Br_1.8_ perovskite films with inhibited photoinduced phase segregation are modified by adding different amounts of 1-butyl-1-methylpiperidinium tetrafluoroborate ([BMP]^+^[BF_4_]^−^) (0.5, 1, 2 and 3 mg mL^−1^) to the CsPbI_1.2_Br_1.8_ precursor solution, and then its effect on the photovoltaic performance of the corresponding devices and perovskite films under light is investigated. The PCE of hole transport layer-free carbon-based perovskite solar cell is improved from 7.13% for a device with a pristine perovskite film to the highest amount of 8.44% for a device with 1 mg mL^−1^ of [BMP]^+^[BF_4_]^−^-modified perovskite film. And the PCE of the device increases first with the increase in the doping concentration of [BMP]^+^[BF_4_] in the perovskite film, but then decreases gradually, and the stability under light is also increased. The modified CsPbI_1.2_Br_1.8_ perovskite film also shows an inhibitory effect on the photoinduced phase segregation phenomenon when compared with the pristine one, with a prolonged time attributed to the reduced phase segregation terminal (pristine: 30.42%@7 min, while the modified: 24.29%@30 min), and the reduced phase segregation rate remaining constant (pristine: *k*_s_ = 1.53 × 10^−2^ s^−1^, while modified one: *k*_s_ = 1.83 × 10^−3^ s^−1^).

## 2. Experimental Section

### 2.1. Chemicals

Cesium iodide (CsI, ≥99.9%) powder was purchased from Sigma-Aldrich (Shanghai) Trading Co. (Shanghai, China). Lead iodide (PbI_2_, ≥99.99%) and lead bromide (PbBr_2_, ≥99.99%) were purchased from Xi’an Yuri Solar Co., Ltd. (Xi’an, China). A tin (IV) oxide colloidal dispersion (SnO_2_, 15 wt% in H_2_O) and dimethyl sulfoxide (DMSO) solution were obtained from Alfa Aesar (China) Chemical Co. (Shanghai, China). Conductive carbon paste was obtained from Shanghai MaterWin New Materials Co., Ltd. (Shanghai, China). Deionized water (DI water) with a resistivity of 18.3 MΩ cm was used in all the preparations. ITO substrates with a sheet resistance of ~15 Ω □^−1^ were purchased from Yingkou OPV tech new energy Co., Ltd. (Yingkou, China). The 1-butyl-1-methylpiperidinium tetrafluoroborate ([BMP]^+^[BF_4_]^−^, C_10_H_22_BF_4_N, 99%) was supported by Aladdin Scientific Corp. (Riverside, CA, USA). All of these were used as received unless otherwise specified.

### 2.2. Preparation of SnO_2_ Precursor

The purchased SnO_2_ colloidal dispersion was diluted with DI water in a mass ratio of 1:5, followed by ultrasonic dispersing for over 3 h before use.

### 2.3. Preparation of Perovskite Precursor

A total of 259.8 mg of CsI, 46.1 mg of PbI_2_ and 330.3 mg of PbBr_2_ (in a molar ratio of 1:0.1:0.9) were dissolved in 1 mL of DMSO. The mixture was then placed on a stirrer and stirred continuously overnight at room temperature. Before use, the resulting transparent yellow solution was filtered through a polytetrafluoroethylene (PTFE) filter with a pore size of 0.45 μm.

### 2.4. Preparation of Perovskite Precursors with Different Mass Concentrations of [BMP]^+^[BF_4_]*^−^*

Various amounts of [BMP]^+^[BF_4_]^−^ powder were added directly to the perovskite precursors with mass concentrations of 0.5, 1, 2, and 3 mg mL^−1^. The mixtures were subsequently dispersed using ultrasonic waves for a duration of over 3 h. Before use, the solutions underwent filtration through a polytetrafluoroethylene (PTFE) filter with a pore size of 0.45 μm.

### 2.5. Device Fabrication

The ITO substrates were carefully cleaned using a foamless eradicator and then ultrasonically cleaned with DI water, acetone and ethanol for 15 min in sequence. Before the deposition of the electron transport layers, they were all dried with high-pressure nitrogen gas and then exposed to a UV/ozone cleaner for 15 min. SnO_2_ electron transport layers were then spin-coated onto the ITO substrates from a SnO_2_ precursor solution. The preparation process of the SnO_2_ precursor solution was listed as follows: After cooling, the samples were transferred to a nitrogen-filled glove box to deposit the perovskite layers using a spin-coating method. Briefly, 100 μL of the perovskite precursor without (pristine) or with different mass concentrations of [BMP]^+^[BF_4_]^−^ (0.5, 1, 2 or 3 mg mL^−1^) was dropped onto the SnO_2_ layer and then spin-coated first at a low speed of 1500 rpm for 20 s and then at a high speed of 5000 rpm for 60 s. The wet perovskite films were then annealed at 35 °C for 15 s and then at 280 °C for 10 min. Finally, compact layers of carbon electrodes were screen printed onto the perovskite films in air, followed by annealing at 120 °C for 15 min on a hot plate. The active area of each electrode was fixed at 0.07 cm^−2^ via screen printing.

### 2.6. Characterization

The top view and cross-sectional morphologies of the samples were characterized using a Quanta 250FEG (FEI Co., Tokyo, Japan) scanning electron microscope (SEM). The crystalline properties of the perovskite films were characterized by an X-ray diffractometer (XRD, SmartLab, Rigaku, Japan) using Cu K*_α_* radiation (40 kV, 30 mA). The scanning speed was 5° per minute at a step of 0.02°. Absorption spectra were obtained using a JASCOV-570 (JASCO., Tokyo, Japan) UV/VIS/NIR spectrometer. *J*-*V* curves were measured in air using a Keithley 2400 source meter (Keithley Instruments, Inc., Solon, OH, USA) under simulated AM 1.5 G irradiation (100 mA cm^−2^), with the illumination source pre-calibrated using a Si reference cell system (91150V, Newport) (Newport Corporation, Irvine, CA, USA). The scan was performed in 0.02 V steps from 1.2 to −0.1 V (reverse scan) with a 0.1 s time delay between each point.

## 3. Results and Discussion

**Figure 1:**
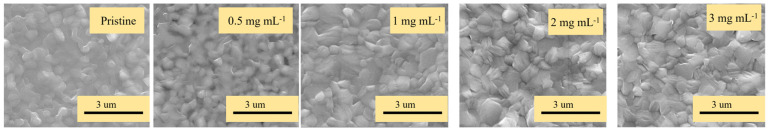
Top view SEM images of pristine CsPbI_1.2_Br_1.8_ and CsPbI_1.2_Br_1.8_ films doped with different mass concentrations (0.5, 1, 2 and 3 mg mL^−1^) of [BMP]^+^[BF_4_]^−^.

In terms of film morphology, CsPbI_x_Br_3−x_ is sensitive to deposition methods, doping and processing conditions such as the spinning speed, environmental temperature and annealing temperature, etc. Figure 1 shows the top view SEM images of the pristine CsPbI_1.2_Br_1.8_ and CsPbI_1.2_Br_1.8_ films doped with different mass concentrations (0.5, 1, 2 and 3 mg mL^−1^) of [BMP]^+^[BF_4_]^−^. It is evident that the surface morphologies of the CsPbI_1.2_Br_1.8_ films doped with different concentrations of [BMP]^+^[BF_4_]^−^ do not differ significantly from the undoped film. All films have a flat surface without any pinholes.

**Figure 2:**
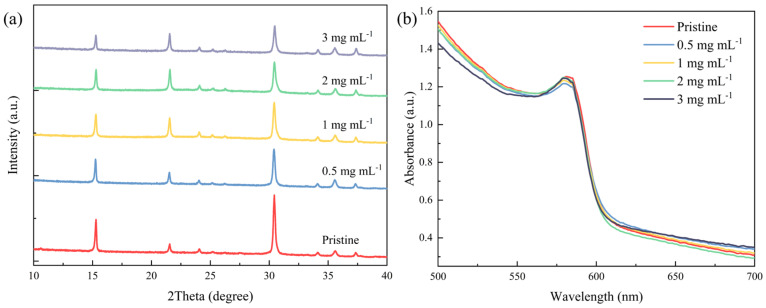
(**a**) XRD pattern and (**b**) absorption spectrum for pristine CsPbI_1.2_Br_1.8_ and CsPbI_1.2_Br_1.8_ films doped with different mass concentrations (0.5, 1, 2 and 3 mg mL^−1^) of [BMP]^+^[BF_4_]^−^.

To establish the impact of the [BMP]^+^[BF_4_]^−^ additive on the crystallinity and photon absorption of the perovskite thin films, an X-ray diffractometer and UV/VIS spectrometer characterization of the pristine and [BMP]^+^[BF_4_]^−^-modified thin films is carried out. The XRD patterns of the pristine and [BMP]^+^[BF_4_]^−^-modified films all exhibit typical perovskite peaks at 2θ = 15.45°, 20.41° and 30.63°, which are assigned to the (100), (110), and (200) lattice planes of the CsPbI_1.2_Br_1.8_ perovskite crystal, respectively [39], as shown Figure 2a. And there are no impurity phases in any of the films. However, the normalized XRD spectra in Figure S1 (Supporting Information) show that the crystalline grains in the [BMP]^+^[BF_4_]^−^-modified CsPbI_1.2_Br_1.8_ film all have a much more preferred (100) orientation. It is speculated that the (100) orientation of the CsPbI_1.2_Br_1.8_ grains is preferentially perpendicular to that of the ITO/SnO_2_ substrate. This orientation is highly advantageous for effective carrier transport and injection in the corresponding devices. Furthermore, the peak intensity of the perovskite film decreases as the mass concentration of the [BMP]^+^[BF_4_]^−^ increases to 3 mg mL^−1^. This suggests that higher mass concentrations may weaken the crystallinity of the modified films with increased scattering from grain boundaries or intragranular defects, which is not beneficial for the photovoltaic performance of the final devices [40]. 

The absorption spectra in Figure 2b indicate that the control film and the [BMP]^+^[BF_4_]^−^-modified CsPbI_1.2_Br_1.8_ films process a similar sharp-cutting absorption edge at ~605 nm corresponding to a bandgap of ~2.05 eV, which is consistent with the values reported in the literature [39,41]. However, this increase in mass concentration leads to a slight decrease in the absorption intensity in the 500–580 nm absorption region, mainly due to the addition of [BMP]^+^[BF_4_]^−^ in the perovskite films.

**Figure 3:**
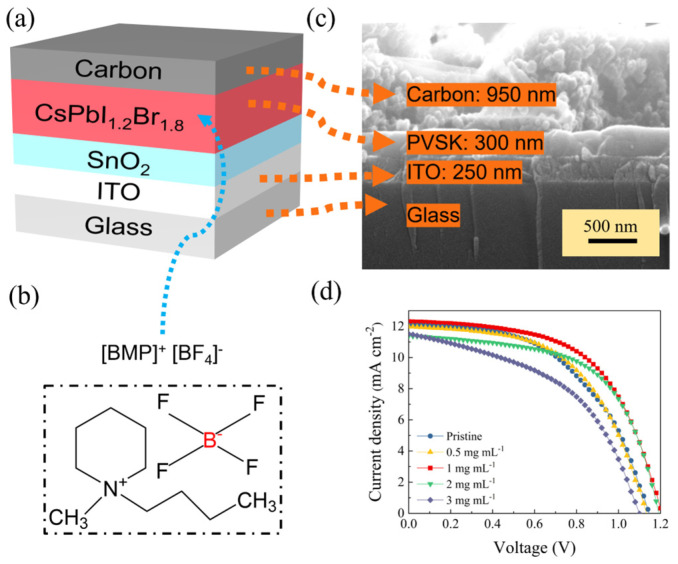
Perovskite solar cell characterization. (**a**) Schematic of the carbon-based all-inorganic perovskite solar cell, (**b**) the chemical structure of [BMP]^+^[BF_4_]^−^, (**c**) cross-sectional SEM image of the full device stack made from CsPbI_1.2_Br_1.8_ with 1 mg mL^−1^ of [BMP]^+^[BF_4_]^−^ as the additive in the perovskite precursor, (**d**) *J*-*V* characteristics of the representative pristine and 0.5, 1, 2 and 3 mg mL^−1^ of [BMP]^+^[BF_4_]^−^-modified CsPbI_1.2_Br_1.8_ devices with the best-performing targets.

The effect of the [BMP]^+^[BF_4_]^−^ additive on the performance of CsPbI_1.2_Br_1.8_ solar cell devices are also investigated. Therefore, we depict the all-inorganic device with a hole transport layer-free architecture (Figure 3a), where SnO_2_ and carbon are used as the electron transport layer and counter electrode, respectively. The cross-sectional SEM image for a representative cell based on 1 mg mL^−1^ of a [BMP]^+^[BF_4_]^−^ (see Figure 3b for the chemical structure)-modified CsPbI_1.2_Br_1.8_ film is shown in Figure 3c (note that the SnO_2_ is too thin to be observed). For comparison, we also prepare pristine and 0.5, 2 and 3 mg mL^−1^ of [BMP]^+^[BF_4_]^−^-modified CsPbI_1.2_Br_1.8_ perovskite solar cells, of which the typical *J*-*V* curves and corresponding parameters for the best-performing target devices are shown in Figure 3d and Table 1, respectively. As we can see, the pristine device exhibited an open circuit voltage (*V*_OC_) of 1.14 V, a short circuit current density (*J*_SC_) of 12.06 mA cm^−2^ and a fill factor (FF) of 0.52, resulting in a PCE of 7.13%. With the increase in the doping concentration of [BMP]^+^[BF_4_] in the perovskite film, the PCE increases first and then decreases gradually. When the doping concentration of the [BMP]^+^[BF_4_] reaches 1 mg mL^−1^, the corresponding CsPbI_1.2_Br_1.8_ device exhibits the highest PCE, that of 8.44%, with a *V*_OC_ of 1.21 V, a *J*_SC_ of 12.32 mA cm^−2^ and an FF of 0.57. In addition, the additions of [BMP]^+^[BF_4_]^−^ to the perovskite light absorber all provide a higher performance compared to that of the pristine one, mainly in terms of the increase in the *V*_OC_ and FF. The optimized addition mass concentration of the [BMP]^+^[BF_4_]^−^ can be determined to be 1 mg mL^−1^.

**Figure 4:**
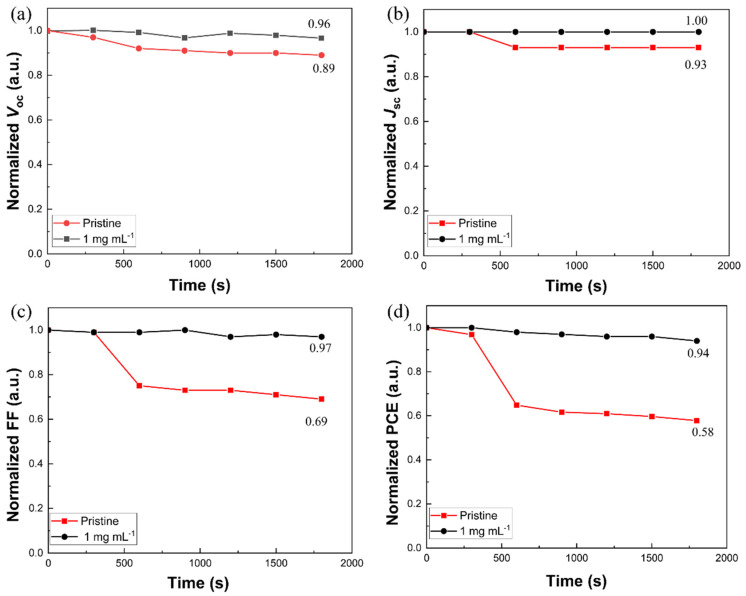
Evolution of the four normalized parameters obtained from the *J-V* curves for the representative pristine and 1 mg mL^−1^ of [BMP]^+^[BF_4_]^−^-modified CsPbI_1.2_Br_1.8_ perovskite solar cells after exposure to different light durations, aged under a simulated air mass and 1.5 sunlight (400 mW cm^−2^) at room temperature in ambient air: (**a**) *V*_OC_, (**b**) *J*_SC_, (**c**) FF and (**d**) PCE.

To further understand the effect of [BMP]^+^[BF_4_]^−^ additives on the performance of CsPbI_1.2_Br_1.8_ perovskite solar cells, the *J*-*V* curves of the pristine and 1 mg mL^−1^ of [BMP]^+^[BF_4_]^−^-modified perovskite solar cells were tested after exposure to different light durations. Figure 4a–d shows the variations in the four normalized photovoltaic parameters (*V*_OC_, *J*_SC_, FF and PCE) of the different devices with respect to the light duration. It can be seen that the four parameters of the pristine device show a significant decrease with the increase in light exposure time, whereas the decrease for that of the 1 mg mL^−1^ [BMP]^+^[BF_4_]^−^-modified device is significantly slower. For example, the PCE of the untreated device gradually decreased from an initial 6.99% to 4.04% after 30 min of light exposure, with a decrease of about 42%, while the PCE of the [BMP]^+^[BF_4_]^−^-treated device gradually decreased from an initial 7.39% to 6.95%, with a decrease of only about 6%. It can be speculated that due to the suppressive effect of the [BMP]^+^[BF_4_]^−^ additive on the photoinduced phase segregation phenomenon of the CsPbI_1.2_Br_1.8_ films, the stability of the perovskite solar cells under light is increased.

**Figure 5:**
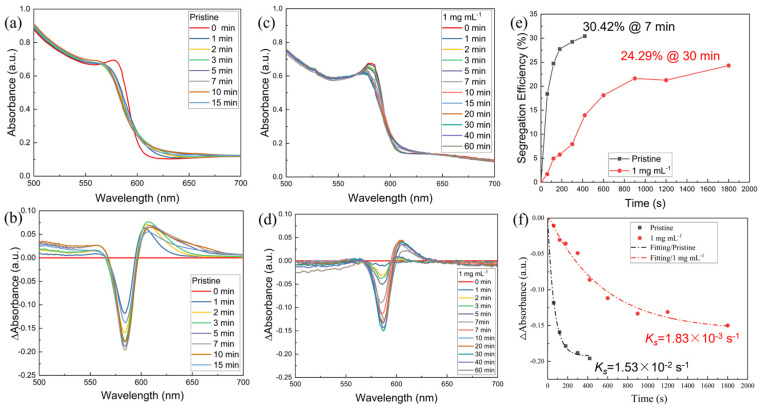
UV-Vis absorption spectra of (**a**) pristine and (**c**) 1 mg mL^−1^ of [BMP]^+^[BF_4_]^−^-modified CsPbI_1.2_Br_1.8_ perovskite films recorded after different light exposure duration times (AM 1.5G 400 mW cm^−2^). (**b**,**d**) Spectra of ΔA values obtained from (**a**,**c**), respectively. (**e**) The phase segregation terminal and (**f**) spectra of ΔA values as a function of the light exposure duration curves obtained from (**a**,**c**). The dashed, doted lines in (**f**) are linear fits to the data using monoexponential decay.

Upon irradiation, Br-mixed halogen perovskites will undergo the internal segregation of halogen ions, resulting in the formation of structural domains that are enriched in either bromides or iodides. To investigate the photoinduced phase separation phenomenon of CsPbI_1.2_Br_1.8_ thin films, we prepared pristine and 1 mg mL^−1^ of [BMP]^+^[BF4]^−^-modified CsPbI_1.2_Br_1.8_ perovskite films. Both films were then subjected to UV-Vis absorption spectroscopy after irradiation for different times using simulated sunlight at 400 mW cm^-2^ AM1.5G. Under this continuous irradiation, the temperature of the films is approximately 40 °C. From the UV-Vis absorption spectra (Figure 5a) of the pristine perovskite film, it can be found that when the light duration was between 0 and 7 min, the intensity of the absorption peak at the wavelength of 584 nm gradually weakened with the increase in light duration, indicating a decrease in the mixed halide content in the perovskite films. The intensities of the absorption peaks at 560 and 620 nm gradually increase, indicating the formation of Br-rich and I-rich phases. As the illumination time is further increased (10 min and 15 min), the intensity of the absorption peaks at 584 nm gradually increases, while the intensity of the absorption peaks at 560 and 620 nm gradually decreases (Figure 5a). This suggests that after the film reaches the phase segregation terminal (30.42%@7 min, Figure 5b,e), the continued strong illumination leads to the phase recovery phenomenon of the perovskite film portion, i.e., the light-induced self-healing phenomenon, which is consistent with a previous report [17]. Similarly, from Figure 5c–e, it can be seen that under continuous light irradiation, the CsPbI_1.2_Br_1.8_ (treated with 1 mg mL^−1^ of [BMP]^+^[BF_4_]^−^) film initially showed a photoinduced phase segregation phenomenon, and the continuous light irradiation led to a photoinduced self-healing phenomenon when the duration time exceeded the limit of the phase segregation terminal (24.29%@30 min, Figure 3d,e). In addition, the curves representing the phase segregation rates were analyzed using a monoexponential decay function, as shown in Figure 5f. The phase segregation rate constant *k*_s_ of the [BMP]^+^[BF_4_]^−^-modified CsPbI_1.2_Br_1.8_ is 1.83 × 10^−3^ s^−1^, while that of the pristine one is 1.53 × 10^−2^ s^−1^. There is an order of magnitude difference between the former and the latter, suggesting that the addition of [BMP]^+^[BF_4_]^−^ could reduce the rate of phase segregation. Thus, we can conclude that under continuous light irradiation, the CsPbI_1.2_Br_1.8_ films will undergo photoinduced phase separation followed by the light-induced self-healing phenomenon upon reaching the limit of the phase separation. And, the addition of [BMP]^+^[BF_4_]^−^ can inhibit the photoinduced phase separation of CsPbI_1.2_Br_1.8_ films.

## 4. Conclusions

Our study mainly focuses on the effect of [BMP]^+^[BF_4_]^−^ additives on the performance of CsPbI_1.2_Br_1.8_ solar cells and their impact on the phenomenon of the photoinduced phase segregation of CsPbI_1.2_Br_1.8_ thin films, paving the way for their use as efficient and light irradiation stable photovoltaic devices. The effects of [BMP]^+^[BF_4_]^−^ additives on the photovoltaic properties and stability of the devices were investigated by preparing hole transport layer-free carbon-based perovskite solar cells, with pristine or 0.5, 1, 2 and 3 mg mL^−1^ of [BMP]^+^[BF_4_]^−^-modified CsPbI_1.2_Br_1.8_ films as the light harvester layers for each device. The results showed that the PCE of the device increases first with the increase in the doping concentration of [BMP]^+^[BF_4_] in the perovskite film, but then it decreases gradually. The device without the addition of [BMP]^+^[BF_4_]^−^ additives in the perovskite shows a PCE of 7.13%, while the device shows the highest PCE of 8.44% with the doping concentration of [BMP]^+^[BF_4_]^−^ reaching 1 mg mL^−1^, and its stability under light increases substantially, which may be related to the significant inhibition of the photoinduced phase segregation process. The impact of the [BMP]^+^[BF_4_]^−^ additive on the photoinduced phase separation of CsPbI_1.2_Br_1.8_ films is investigated using UV-Vis absorption spectra and the corresponding difference in absorption spectra as a function of the light exposure time at room temperature. The photoinduced phase segregation limit time of the CsPbI_1.2_Br_1.8_ films increased from 7 to 30 min after the addition of 1 mg mL^−1^ of the [BMP]^+^[BF_4_]^−^ additive. This indicates that the [BMP]^+^[BF_4_]^−^ additive could effectively reduce the phase segregation phenomenon of the CsPbI_1.2_Br_1.8_ films. The photoinduced phase segregation phenomenon of CsPbI_1.2_Br_1.8_ films is suppressed by the [BMP]^+^[BF_4_]^−^ additive. 

## Figures and Tables

**Table 1:** *J*-*V* parameters of the representative devices according to Figure 3d.

Devices	*V*_OC_/V	*J*_SC_/mA cm^−2^	FF	PCE/%
Pristine	1.14	12.06	0.52	7.13
0.5 mg mL^−1^	1.13	11.97	0.54	7.36
1 mg mL^−1^	1.21	12.32	0.57	8.44
2 mg mL^−1^	1.19	11.39	0.59	8.03
3 mg mL^−1^	1.20	11.58	0.58	8.02

## Data Availability

Data are contained within the article and Supplementary Materials.

## Supplementary Materials

The following supporting information can be downloaded at: https://www.mdpi.com/article/10.3390/molecules29071476/s1, Figure S1. Normalized XRD patterns of pristine CsPbI1.2Br1.8 and CsPbI1.2Br1.8 films doped with different mass concentrations (0.5, 1, 2 and 3 mg mL-1) of [BMP]+[BF4]-.
