# Supplementary material for: [BMP]+[BF4]−-Modified CsPbI1.2Br1.8 Solar Cells with Improved Efficiency and Suppressed Photoinduced Phase Segregation
Source: Molecules. 2024 Mar 26;29(7):1476. doi: 10.3390/molecules29071476 (PMC11013225; doi:10.3390/molecules29071476)
Supplement: Supplementary file 1 [file molecules-29-01476-s001.zip › molecules-2875725-supplementary.pdf]

Supporting Information

$[BMP]^+[BF_4]^-$-Modified $CsPbI_{1.2}Br_{1.8}$ Solar Cells with Improved Efficiency and Suppressed Photoinduced Phase Segregation

Haixia Xie[1, 2, *], Lei Li[3], Jiawei Zhang[3], Yihao Zhang[1], Yong Pan[1], Jie Xu[1], Xingtian Yin[3, *], Wenxiu Que[3]

[1]School of Science, Xi'an University of Architecture and Technology, Xi'an 710055, People’s Republic of China

[2]State Key Laboratory for Strength and Vibration of Mechanical Structures, School of Aerospace Engineering, Xi'an Jiaotong University, Xi'an 710049, People's Republic of China

[3]Electronic Materials Research Laboratory, Key Laboratory of the Ministry of Education, International Center for Dielectric Research, Shaanxi Engineering Research Center of Advanced Energy Materials and Devices, School of Electronic Science and Engineering, Xi’an Jiaotong University, Xi’an 710049, People’s Republic of China

*Correspondence: xiehaixia@xauat.edu.cn (H. X), xt_yin@xjtu.edu.cn (X. Y)

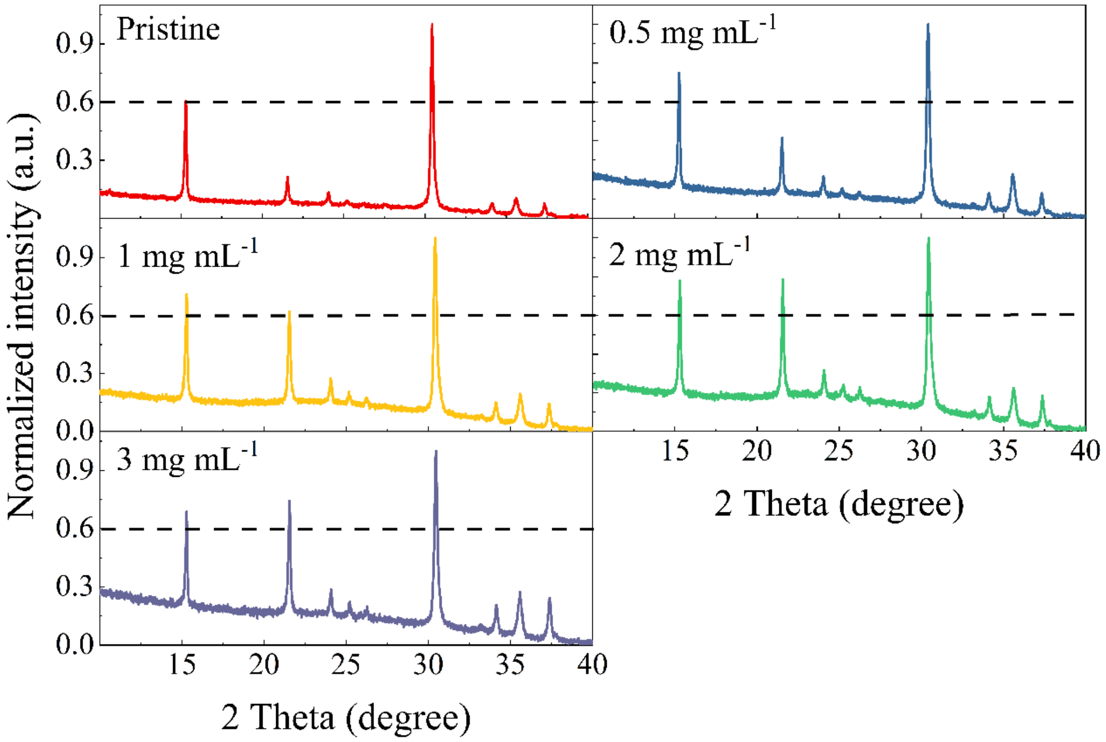

Figure S1. Normalized XRD patterns of pristine $CsPbI_{1.2}Br_{1.8}$ and $CsPbI_{1.2}Br_{1.8}$ films doped with different mass concentrations (0.5, 1, 2 and 3 mg mL^{-1}) of $[BMP]^{+}[BF_4]^{-}$.
